# Study on dust migration law and spray dedusting technology in parallel double belt transportation

**DOI:** 10.1038/s41598-022-09200-1

**Published:** 2022-03-30

**Authors:** Deji Jing, Zhuo Jiang, Mingxing Ma, Tian Zhang, Hongwei Liu, Tao Yu

**Affiliations:** 1 School of Safety Science and Engineering, Liaoning Technical University, Fuxin, 123000 Liaoning China; 2 Key Laboratory of Mine Thermal Power Disasters and Prevention, Ministry of Education (Liaoning Technical University), Fuxin, 123000 Liaoning China

**Keywords:** Energy science and technology, Engineering

## Abstract

To effectively solve the problem of dust pollution caused by the parallel double-belt transportation of coal in a coal preparation plant, taking the Huangyuchuan coal preparation plant as an example, a numerical model of the air flow-dust distribution was established by means of simulation. The flow lines between the strips of tape and the tail of the tape machine will gather, and there will be backflow on the right side of the 3001 tape and left side of the 3002 tape. Under the action of wind current, most of the dust particles larger than 10 μm are distributed in the range of 0–5 m on both sides of the tape; dust particles smaller than 10 μm spread to the entire preparation workshop. Combined with field test verification, dust pollution is mainly concentrated at the guide trough, the feed inlet, the rear of the machine, and the joint of the belt corridor. Based on this, a targeted spray dust reduction treatment plan is proposed. By measuring the dust concentration before and after the treatment of dust-polluted areas, it is proven that the dust reduction efficiency of this plan can reach more than 90%.

## Introduction

According to the statistics in China’s 2020 Coal Industry Development Annual Report, the national coal production in 2020 was 3.90 billion tons, representing an increase of 1.4% year-on-year. It is estimated that China’s raw coal output will reach 3.91 billion tons in 2021, indicating that coal is still an important resource consumed in the country. As an indispensable area in the coal industry, coal preparation plants are the most severely polluted and difficult-to-treat areas of respirable dust. Working in places with high concentrations of respirable dust for a long time will cause great harm to human health^1–3^; workers in other occupations are more likely to suffer from bronchitis and pneumoconiosis, and in severe cases, cancer^4^. At the same time, high-concentration dust can cause dust explosion accidents^5^ when exposed to open flames, which poses a threat to life and health. The dust pollution problem of coal preparation plants mainly comes from the process of coal transportation by belts, so it is urgent to solve the problem of dust pollution during belt transportation in coal preparation plants.

Regarding the problem of dust pollution, many scholars have conducted in-depth research on the law of dust escape and treatment plans. For example, Zhou^6^ and others designed a suspended mine dust filtering system to solve the problem of high dust concentration in a coal mine roadway which is difficult to solve by traditional dust removal methods. Xiu^7^ and others studied the dust diffusion law under different air flow rates and proposed a comprehensive dust control plan. Ma^8^ and others studied the mechanism of coal dust precipitation and diffusion and proposed a closed negative pressure dust reduction technology for coal conveying belt systems. Zhang^9^ et al. studied the law of dust and the distribution of dust concentration during the operation of a belt conveyor and proved the rationality of the Euler-Lagrangian method to simulate the distribution of dust in the roadway. Nie^10–12^ et al. studied the dust diffusion law through the CFD-DPM airflow coupling method. Du^13,14^ et al. used a computational fluid dynamics (CFD) model to study the law of dust movement and proposed corresponding control measures. Fang^15^ and others proposed a two-phase air–water dust reduction spray technology and concluded that the dust catching stage of the fine water mist of different particle sizes is different. Sun^16,17^ et al. studied the agglomeration and removal characteristics of fine particles under different turbulent flow fields through experiments and the results showed that small-scale and three-dimensional vortex turbulent agglomeration devices have the best effect. SChaulya^18^ and others combined nozzles and sensors and other equipment to develop an automatic dry mist and dust suppression system. Maksim Mezhericher^19–22^ and others studied gas jets to decompose a thin liquid film to atomize a liquid, and the liquid was atomized into micron and submicron droplets. Yao^23^ analyzed the influence of nozzle parameters on dust reduction efficiency by comparing the advantages and disadvantages of different types of nozzles.

From the research situation of domestic and foreign scholars, if wet dust removal adopts methods such as increasing the spray pressure and reducing the aperture to improve atomization efficiency, although certain results are obtained, only the working conditions and parameters are optimized and the potential for improved atomization efficiency is limited. This article uses the point-type dust removal method. After analyzing the dust pollution law of a parallel double belt conveyor during transportation, for several areas with serious dust pollution, a wet dust removal scheme is designed without affecting the coal quality. The supersonic water pumping siphon innovative atomization nozzle achieves the purpose of precise and efficient dust removal.

## Numerical simulation of the preparation workshop

**Figure 1:**
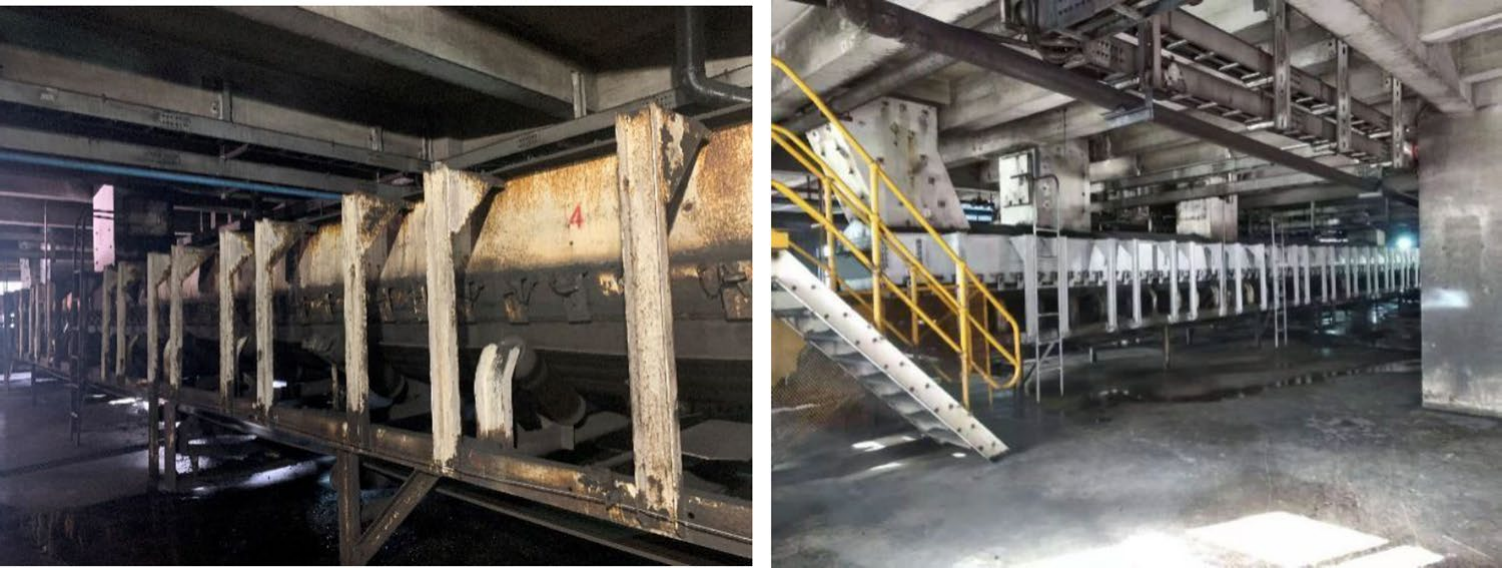
Site map of the preparation workshop.

This paper studies the Huangyuchuan coal preparation plant located in the southwest of the Zhungeer Coalfield in Inner Mongolia. The coal preparation plant has 7 floors. With the help of dust sampler, the dust concentration sampling survey is conducted on the first floor of the preparation workshop around belts 3001, 3002, and 3003 and from the 2nd to the 7th floor. First, put the new weighed filter membrane into the sampling head of the dust sampler, start the instrument when testing at the measuring point, set the sampling time of 5 min, and the instrument will stop automatically after sampling, and measure the dust concentration according to the weight gain of the filter membrane. Then, the dust dispersion degree is measured by the filter membrane dissolving smear method with the help of a microscope. After the dust concentration and dispersion are measured, the results show that the total dust concentration and respirable dust concentration of the working place on the first floor of the coal preparation plant seriously exceed the standard, i.e., the total dust concentration(ie total concentration) ranges from 90 to 180 mg/m^3^, the highest concentration can reach 180 mg/m^3^, and the concentration of respirable dust is 13–57.3 mg/m^3^. Among the total dust, the dispersion test results show that < 5 μm dust, i.e., the most harmful to humans, accounted for 49–75%, 5–10 μm dust accounted for 15–32%, 10–20 μm dust accounted for 8–15%, and > 20 μm dust accounted for 2–6%. The dust deposits on the work site are relatively thick. If they are not cleaned up in time, they will cause secondary dust pollution under the influence of people walking and wind flow disturbance. Because 3003 tape transports fine coal, the dust concentration is low and does not need to be treated. The preparation workshop site is shown in Fig. 1. To study the dust particle diffusion law in depth, two tapes, i.e., 3001 and 3002, are selected as models for simulation analysis.

### Establishment of the mathematical model

Air flow is an important factor leading to dust pollution^24,25^. This article first establishes a wind flow field and then simulates and studies the dust emission law through the coupling of dust particles and the wind flow field. The wind flow in the belt transportation area in the workshop is mostly unstable.

Turbulent kinetic energy *k* transport equation:

1 $$\rho \frac{\partial k}{{\partial t}} + \rho u \cdot \nabla k = \nabla \left\{ {\left( {\mu + \frac{{\mu_{T} }}{{\sigma_{k} }}} \right)\nabla {\text{k}}} \right\} + P_{k} - \rho \varepsilon$$

Turbulent energy dissipation rate *ε* transportation equation:

2 $$\rho \frac{\partial \varepsilon }{{\partial t}} + \rho u \cdot \nabla \varepsilon = \nabla \left\{ {\left( {\mu + \frac{\partial T}{{\partial \varepsilon }}} \right)\nabla \varepsilon } \right\} + C_{\varepsilon 1} \frac{\varepsilon }{k}P_{k} - C_{\varepsilon 2} \rho \frac{{\varepsilon^{2} }}{k}$$

Turbulent viscosity coefficient *μ*_*T*_:

3 $$\mu_{T} = \rho C_{\mu } \frac{{k^{2} }}{\varepsilon }$$

In the formulas, *t* is time, s; *ρ* is the gas density, kg/m^3^; *k* is the turbulent kinetic energy, m^2^/s^2^; *ε* is the turbulent energy dissipation rate, m^2^/s^3^; *P*_*k*_ is the turbulent kinetic energy generation term caused by the average velocity gradient; *μ*_*T*_ is the turbulent viscosity coefficient, Pa·s; *Cμ, Cɛ*_*1*_, and *Cɛ*_*2*_ are empirical constants, i.e., *Cμ* = 0.09, *Cɛ*_*1*_ = 1.44, and *Cɛ*_*2*_ = 1.92; and *σk* = 1.0 is the turbulent Prandtl number of the *k* equation. The simulation of dust particles is based on the particle force in the Lagrangian coordinate system to solve the particle motion trajectory. The particle motion balance equation is expressed in the Cartesian coordinate system as:

4 $$\frac{{du_{p} }}{dt} = F_{D} \left( {u - u_{p} } \right) + \frac{{g_{x} \left( {\rho_{p} - \rho } \right)}}{{\rho_{p} }} + F$$

$F_{D} = \left( {u - u_{p} } \right)$ is the drag force per unit mass of dust particles; *u* is the continuous phase velocity, m/s; *u*_*p*_ is the particle speed, m/s; *μ* is the molecular viscosity coefficient of the fluid, Pa·s; and *ρ*_*p*_ is the density of the fluid and particles, kg/m^3^.

5 $$F_{D} = \frac{18\mu }{{\rho_{p} D_{p}^{2} }}\frac{{C_{D} Re}}{24}$$

### Establishment of the geometric model and meshing

**Figure 2:**
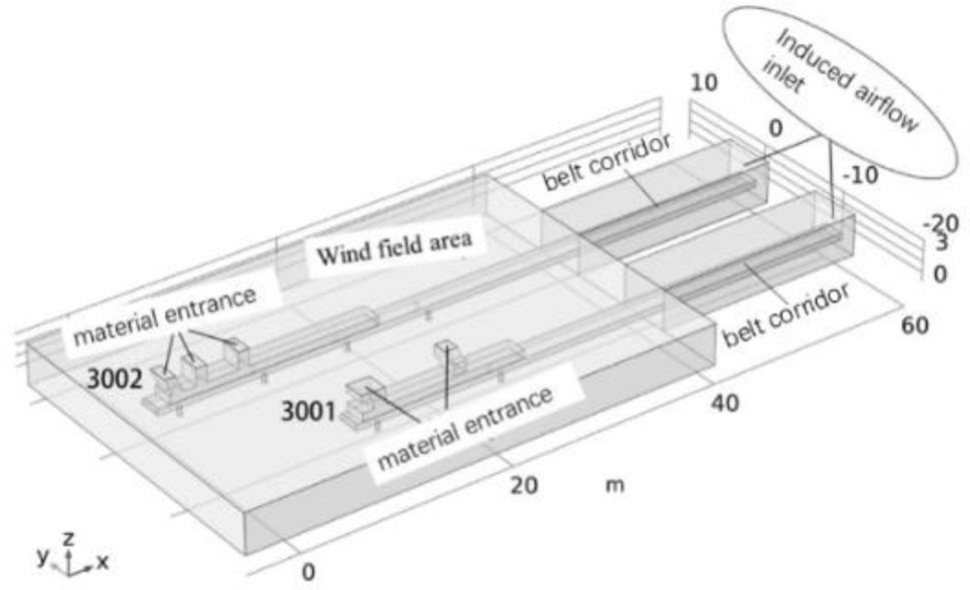
^26^Geometric modeling of the wind flow field.

**Figure 3:**
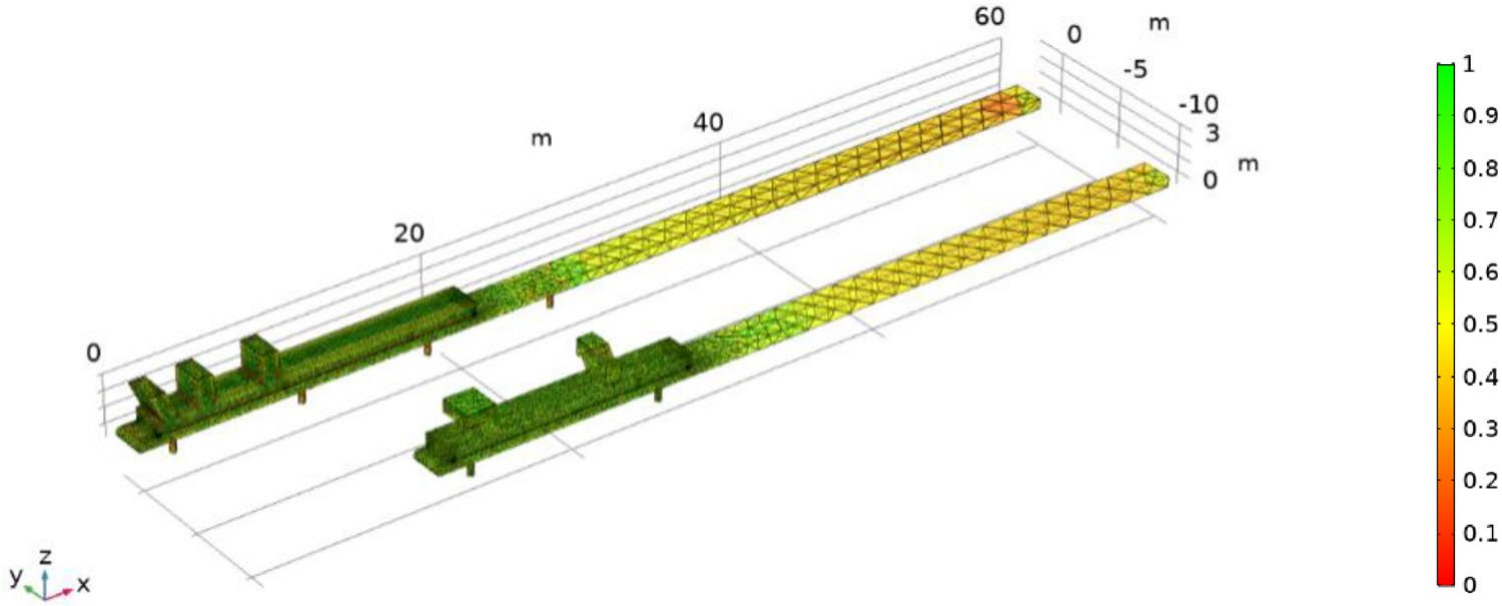
^26^Double tape mesh generation.

**Figure 4:**
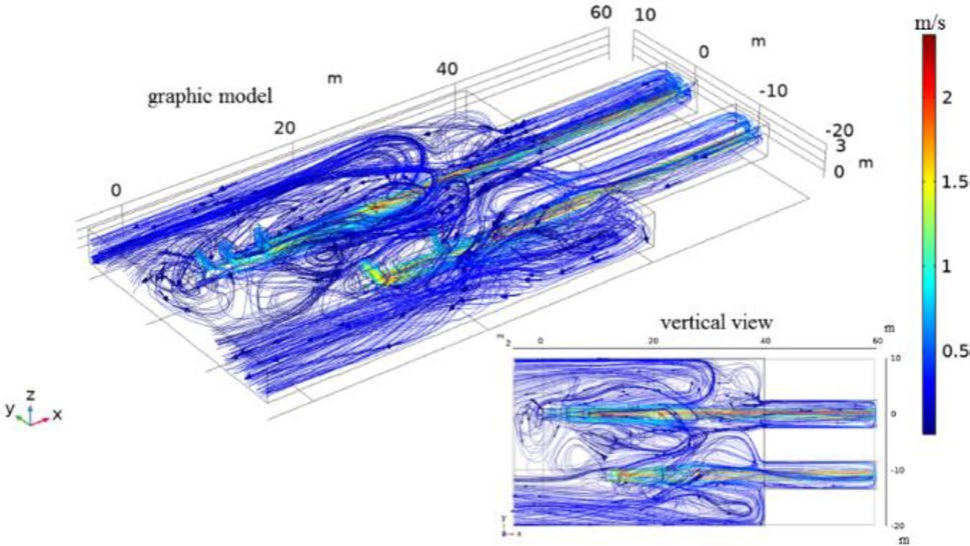
^26^Flow line diagram of the double tape wind flow field.

**Figure 5:**
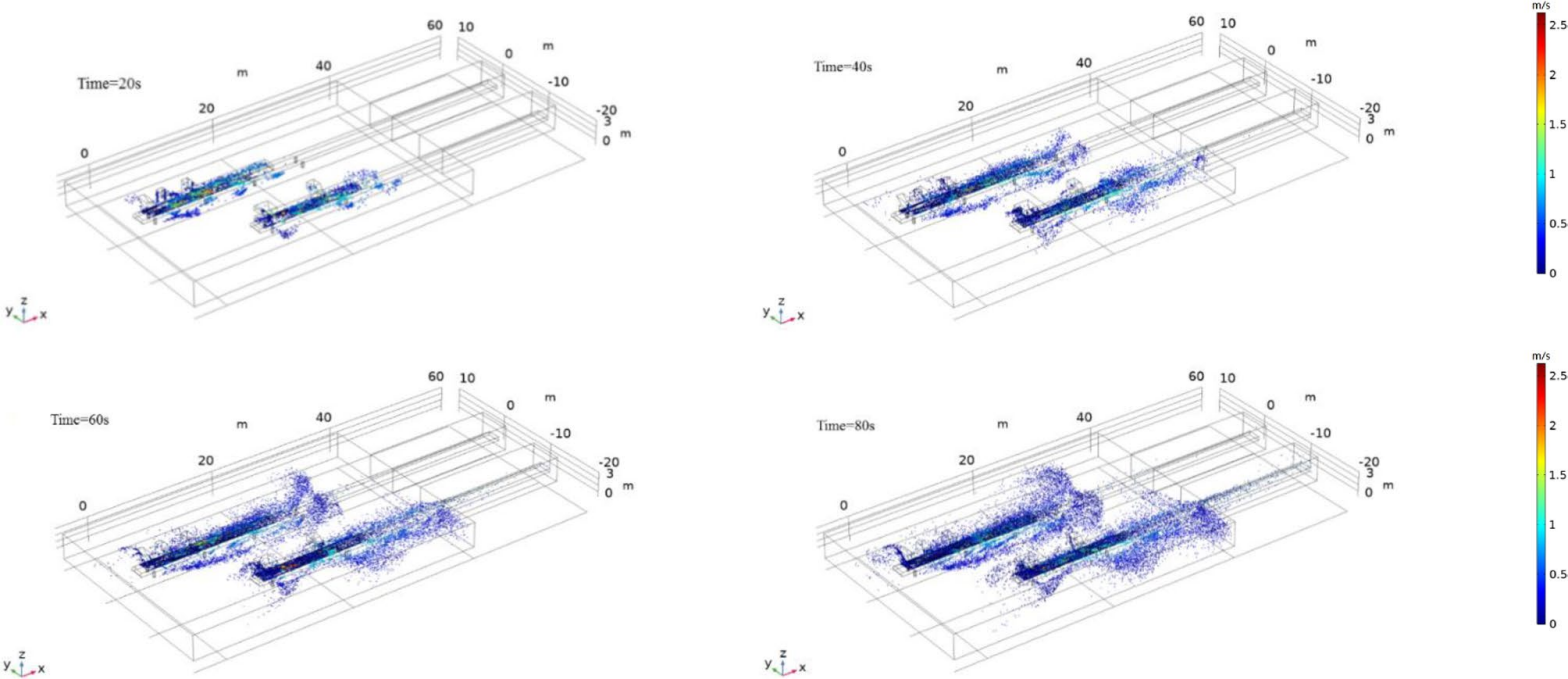
^26^Particle diffusion diagram at different moments in the preparation workshop.

**Figure 6:**
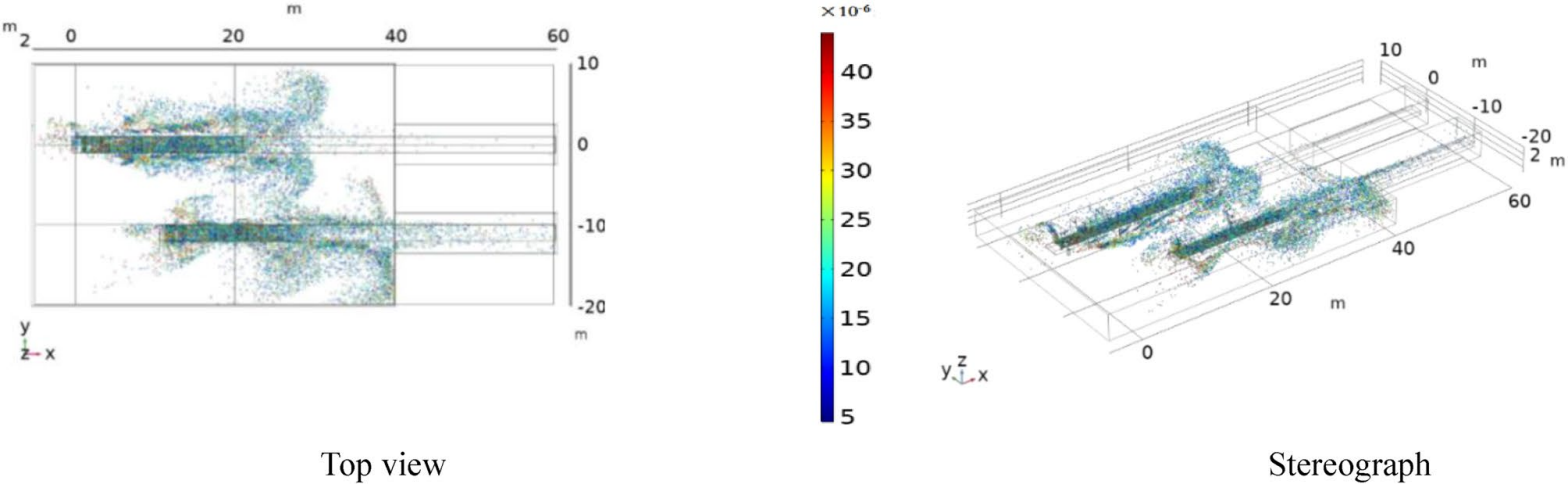
^26^Particle size distribution diagram.

**Figure 7:**
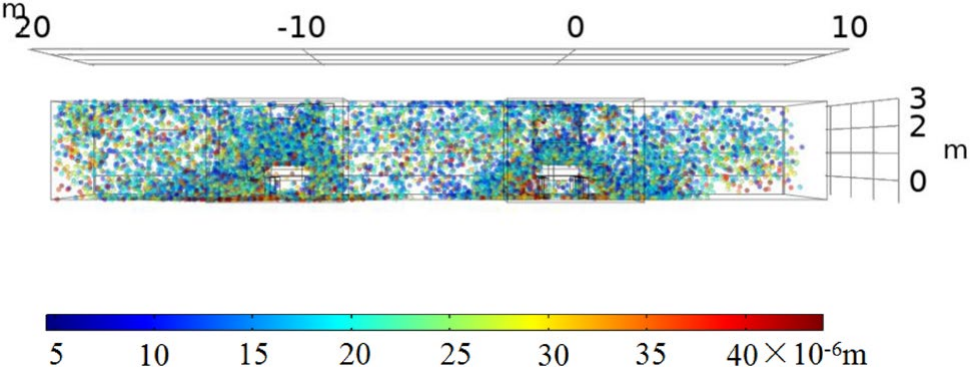
^26^Cross-sectional view of the particle size distribution.

Taking the first floor of the preparation workshop as the research object, the workplace is an indoor space. During geometric modeling, the space of the first floor is approximated as a rectangular area, and the size is established according to its actual size. The length, width and height of the preparation workshop are 60 m, 30 m and 3.8 m, respectively. The 3002 working area on the first floor (not including the belt corridor) is 38.5 m long and the lower belt is 0.8 m high from the ground; the length, width, and height of the guide trough are 20 m, 2 m, and 0.8 m, respectively. The 3001 working area on the first floor (not including the belt corridor) is 28 m long, the distance between the upper and lower belts is 0.6 m, and the height of the lower belt is 0.8 m from the ground; the length, width, and height of the guide trough are 15.6 m, 2 m, and 0.8 m, respectively. The work area is connected to the tape corridor and leads to other areas. Figures 2, 3, 4, 5, 6 and 7 are all completed by COMSOL Multiphysics 6.0^26^ (http://www.comsol.com), and the geometric model of the two tapes and the surrounding wind flow field is established as shown in Fig. 2.

Grid division has an important influence on the simulation results. In this study, three different grid numbers are selected for simulation, represented by grid A, grid B, and grid C, which are composed of 334,281, 651,279, and 1,018,675 grids, respectively. After comparison, the results of grid B and grid C are almost similar, indicating that increasing the number of grids after reaching the appropriate number of grids has no significant impact on the results. Moreover, the number of grid C is large, and the computer needs higher configuration and the simulation operation needs longer time. Grid A and grid B show similar trends, but the absolute values are different, and the simulation results of grid A are rough and can not achieve better accuracy. Taking into account the simulation efficiency, computer configuration and the accuracy of the simulation results, grid B is selected for simulation research^27^. Grid B has no negative value and good grid quality, including tetrahedral number 341,670, triangle 65,712, edge element 3516, vertex element 232. The meshing is shown in Fig. 3.

### Basic parameter setting

There are two air inlets: inlet 1: five inlets, which are also particle release inlets; inlet 2: the wall at the far right end of the tape corridor is also a particle outlet at the same time, and the air outlet is the left wall of the belt conveyor. The model wall conditions are all frozen, and dust particles do not rebound. The specific model parameters and boundary conditions are shown in Table 1.

**Table 1:** Solution model parameters and boundary condition settings.

Parameter name	Parameter setting
Inlet 1 wind speed/(m/s)	0.56
Inlet 2 wind speed/(m/s)	0.08
Air density/(kg/m^3^)	1.225
Gas molecule diffusion coefficient/(m^2^/s)	2 × 10^–5^
Entrance particle number/piece	1000
Temperature/K	293.15
Wall movement/(m/s)	3.27

## Numerical simulation calculation and result analysis

### Wind flow field simulation results and analysis

According to the parameters and boundary conditions described in Chapter 2, “Numerical simulation of the preparation workshop” section, calculated by simulation software, the airflow streamline distribution during the operation of the double belt is shown in Fig. 4. The figure shows that the air flow line moves from the inlet to the belt corridor. The air flow velocity is generally below 0.6 m/s, and the flow line velocity close to the tape can reach more than 1 m/s. The airflow of the two tapes affects each other in the migration process and is hindered and rebounded by the wall at the same time. An airflow streamline accumulation area will be formed between the two tapes and at the tail of the tape machine. Reflux will occur on the right side of 3001 tape and on the left side of 3002 tape.

The 3001 tape is shorter, and the kinetic energy loss during the migration process is small. When it moves to the tape corridor junction, it is hindered and bounced by the wall, causing backflow and forming a streamline gathering area, which is distributed on the right side of the tape. Similarly, during 3002 tape operation, due to the tape distance being long and the loss of airflow kinetic energy by the resistance of air, the speed is not fast enough to continue to move forward. The wind flow line escapes to the left side of the tape at a position approximately 10 m away from the entrance of the tape corridor, forming a return flow. The escaping air flow in the gap of the guide groove of the 3001 belt conveyor and the escaping air current in the gap of the guide groove of the 3002 belt conveyor meet and interact to form a streamline gathering area between the two tapes. The gathering area at the tail of the belt conveyor is affected by the return of the secondary air flow, forming a streamline gathering area.

### Simulation results and analysis of dust particle migration

According to the set boundary conditions, the five inlets of the two tape guide grooves are the particle release entrance, and the particle exit is at the end wall of the rightmost end of the tape corridor. The particle migration trajectory is obtained after an 80 s calculation by the simulation software. Figure 5 shows the particle diffusion simulation results at 20 s, 40 s, 60 s, and 80 s.

After the dust particles fall on the tape, most of the dust particles move with the tape at first, and a small part of the dust particles escape from the inlet or through the gap of the guide groove of the tape conveyor driven by air and are induced by the airflow. With a positive pressure, the movement speed of particles is generally below 0.6 m/s, and the movement speed of particles close to the belt is faster, reaching more than 1 m/s. When the dust particles move to the belt corridor, they are affected by the resistance of the wind flow, and the speed of the dust particles ejected from the inlet gradually decreases. Over time, a large number of dust particles adhered to both tapes.

After the dust particles ejected from the inlet of the 3001 tape conveyor hit the wall of the tape corridor connection, most of them are bounced back, and under the influence of the wind flow field, they escape to the wall side at the tape corridor connection and gather in a large amount. The 3002 tape dust particles have a long migration distance and large kinetic energy loss. In the process of moving in the direction of the tape corridor, the effect of resistance and the influence of backflow cause most of the particles to suspend at 10 m from the entrance of the tape corridor and escape to the side. Small particles of dust are dispersed with the airflow to the space on both sides of the tape. Some deposited dust particles are affected by the secondary air flow and will be suspended again at the tail of the belt conveyor. However, the wind speed at the tail of the belt is relatively high, and the particles diffuse to the surroundings.

It can be seen from Fig. 6 that most of the dust particles larger than 10 μm are distributed in the range of 0–5 m on both sides of the tape; some are attached to the guide groove and the tape, and others are suspended in the air. Dust particles smaller than 10 μm have the most diffusion range. It can be seen from Fig. 7 that small blue dust particles fill most of the space and are difficult to settle when suspended in the air. The diffusion range is 0–10 m on both sides of the tape, while large dust particles settle naturally.

## Field application

When the breathing dust on the scene moves, the air resistance is small and it is easily spread; it easily follows the airflow trajectory and streamline movement, and it is difficult to eliminate by gravity. In the spray dust reduction scheme, the supersonic water suction siphon atomizing nozzle independently developed by the dust team of Liaoning Technical University^28^ is used. When the nozzle is working, the water consumption flow is between 90 ~ 150 mL/min, and the air consumption is 4.0–4.5 m^3^/h. The water consumption and air consumption are both small, and the specific spray effect of the nozzle is tested by the following experiment.

### Experimental research on the spray effect of the nozzle

#### Main experimental equipment

**Figure 8:**
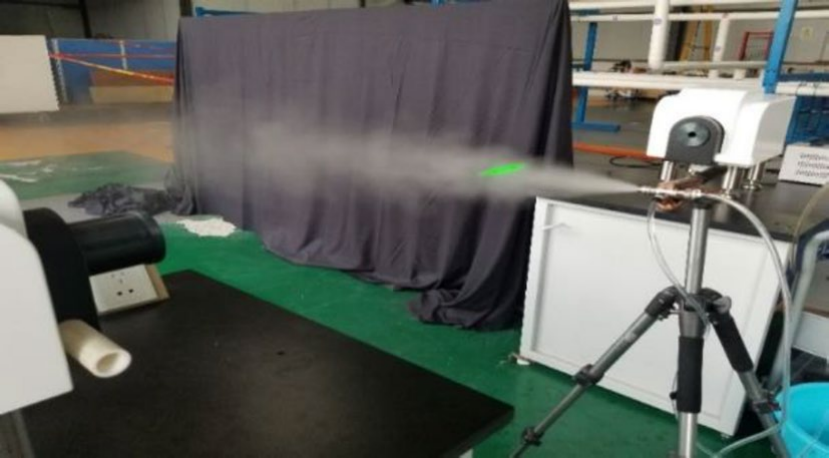
Sprinkler experimental platform.

The experimental platform built by a laser particle size analyzer, air compressor, supersonic siphon atomization nozzle, PVC tube, computer and tripod is shown in Fig. 8.

#### Experimental principle

The principle of information optics is used to analyze the particle size distribution of a particle group by measuring its scattering spectrum.

#### Experimental process and results

**Figure 9:**
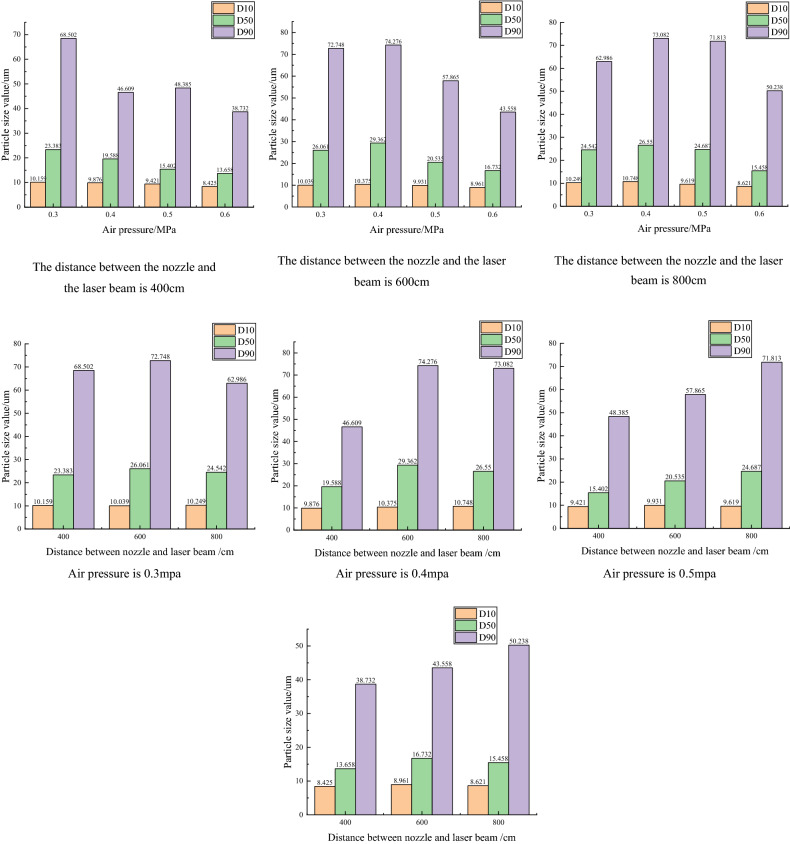
^30^Particle size values under different air pressures and distances.

The experimental equipment is connected, and the laser emission unit and laser receiving unit of the laser particle size analyzer are installed on the same level. The power is turned on, the hood is opened on the side of the receiving area of the instrument, and the laser emitted from the center hole of the detector array is checked. The instrument is adjusted until a uniform and regular green spot is formed in the distance before starting the test. The analyzer needs to perform the background test first and then perform the energy spectrum test. In the experiment, the water absorption height was controlled at 13 cm, and the distance between the nozzle and the laser beam and the air pressure were adjusted. The distance between the nozzle and the laser beam was controlled at 400 cm, 600 cm and 800 cm, and the air pressure was controlled at 0.3 MPa, 0.4 MPa, 0.5 MPa and 0.6 MPa. The values of the three atomization performance evaluation indexes, D10, D50 and D90^29^, were recorded, in which D50 was also called the median diameter. Through OriginPro 2018c^30^ (http://www.OriginLab.com), the data were collated to obtain Fig. 9, and finally analyzed. Experimental data show that the particle size is less than 100 µm. The smaller the particle size is, the more adequate the contact with dust particles. The data of D10 does not change significantly. Comparing D50 and D90, it can be seen that the particle size decreases as the air pressure increases. As the distance increases, the particle size tends to decrease.

### Construction of the spray dust suppression system and installation of the spray equipment

According to the simulation results, dust pollution is mainly concentrated at the guide trough, the tail of the belt conveyor, and the connection of the belt corridor and the inlet. After on-site measurement, comparing the dust concentration in different areas, the results show that the on-site dust-contaminated area is basically consistent with the simulation result, so the simulation is correct. The concentration of respirable dust in these areas is 6.5 to 28.65 times the safety standard, and the dust concentration seriously exceeds the standard. According to the simulation results, a treatment plan is proposed for several areas with serious dust pollution.

**Figure 10:**
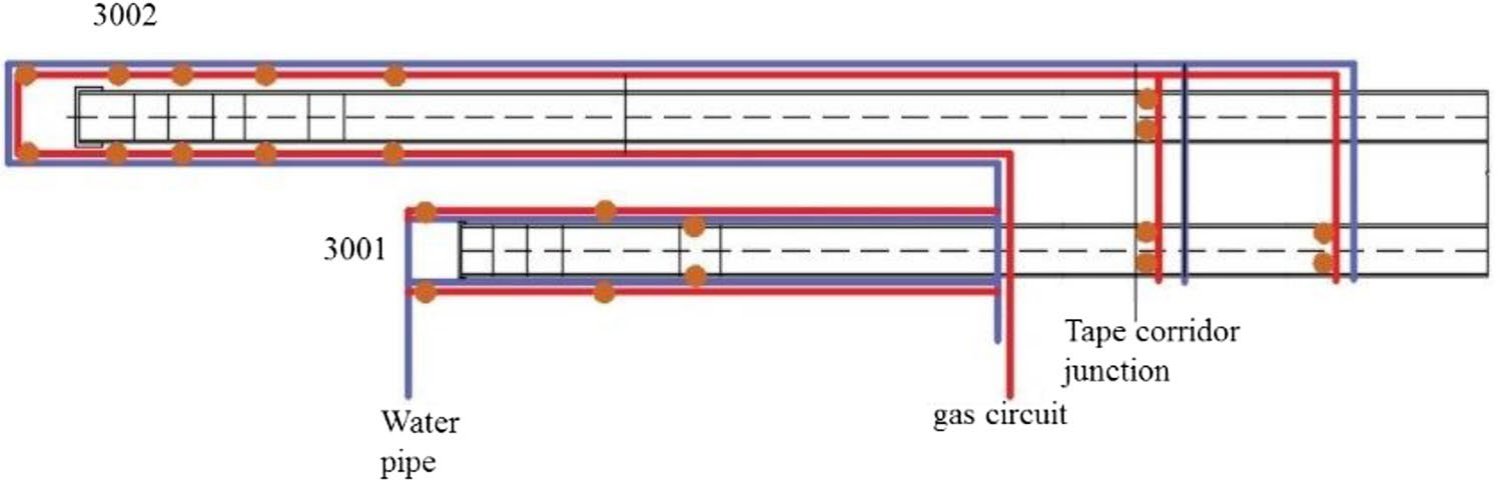
^31^Layout plan of the tape nozzle.

When the system is in production, the lump coal falls on the 3002 tape through the feed port. After a series of processes, such as impact, friction, and crushing, the lump coal will generate a large amount of coal dust, which will spread to the surroundings under the action of the wind flow field. At the same time, to reduce the impact of wet dust suppression on the calorific value of lump coal and to prevent belt slippage, large-flow direct injection must be avoided. Therefore, the layout of the two tape nozzles is shown in Fig. 10. Drawing by AutoCAD 2018^31^ software (http://www.autodesk.com), the orange circle represents the nozzle, the red pipe represents the air path, which needs to be connected to the air compressor and the air tank, and the blue pipe represents the water path.

The layout plan of the inlet is as follows: starting from the vicinity of the first inlet, two rows of 8 supersonic suction siphon atomizing nozzles are arranged in parallel along the two sides of the guide groove of the 3002 belt conveyor. The distance increases successively, and the height is slightly higher than the top of the guide trough. While controlling the dust at the feed inlet, the fog curtain effect also reduces the dust concentration at the guide trough. The nozzle is 2.3 m away from the ground, and the angle is 45° with the pipeline. The air pressure is 0.5 MPa, and the spraying direction is in the direction of the coal flow conveyed by the belt. In the working state, the four oblique fog curtains can cover the coal flow along the direction of the belt movement to reduce the dust concentration.

**Figure 11:**
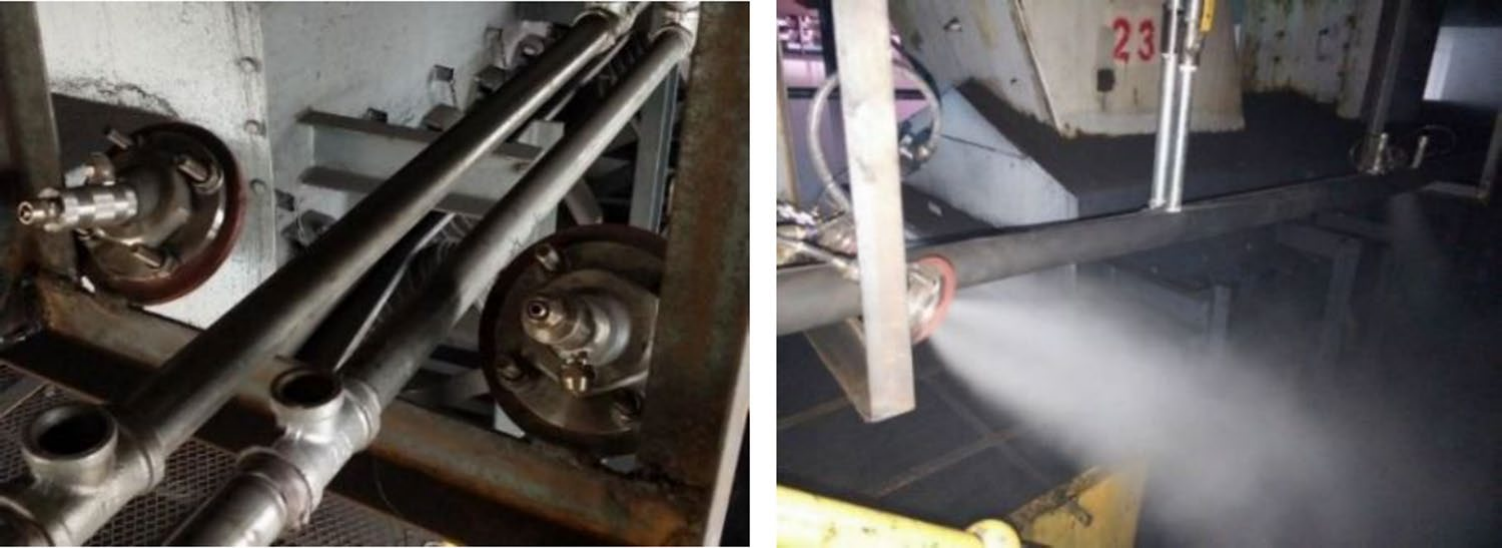
The layout details of the tail nozzle and the atomization effect.

Six nozzles are arranged at the tail of the 3001 belt conveyor, which are located on the left, right, and front sides of the belt, with two on each side, which is suitable for the site conditions. The nozzles on the left and right sides are arranged symmetrically. There are two nozzles arranged on the side with a distance of 5 m between the front and rear. One covers the direction of the airflow to cover and reduce dust, and the other is approximately 45° to the belt conveying direction to pull the surrounding dust. A fog screen is formed on the left and right sides to cover the left and right sides of the rear of the machine, and the front side adopts a positive pressure fog screen covering arrangement that maintains a certain distance from the rear of the machine. Two nozzles are arranged at a distance of 1.5 m from the tail of the 3002 belt conveyor. The mist sprayed by the nozzles is sprayed along the edge of the tail cover to both sides of the tail, covering all surrounding areas. The nozzle air pressure at the tail is controlled within 0.4–0.5 MPa. In time, the governance effect is good. Figure 11 shows the local details of the nozzle arrangement at the rear of the machine and the actual spray effect on site.

The dust at the junction of the tape corridor is mainly concentrated on the 3001 tape. In the layout, two nozzles are installed at the junction of the 3001 and 3002 tape corridors. At the same time, two nozzles are arranged at the junction of the 3001 tape corridor at a distance of 3–4 m. The tape is approximately 1–1.5 m, the air pressure is 0.4 MPa, and the spray direction is vertically downward to spray the tape directly. To avoid large areas wetting the coal and tape, a low-pressure atomization method with low water flow and low humidity is adopted. Field experiments show that the aerosol completely covers the coal flow in the space above the coal flow, forming a relatively static state of motion in the same direction as the coal flow, effectively suppressing the phenomenon of coal dust flying during the migration of coal flow.

### Comparison of the dust removal effect

After installation and commissioning of the spray dust suppression equipment, trial operation is conducted. Several points with serious dust pollution are selected, and each point is sampled three times through the dust sampler. Each sampling time is five minutes, and the dust concentration is measured when the system is running and not running. A breathing zone height of 1.5 m is selected, the sampled filter membrane is brought back to the laboratory for weighing and calculation of the average of the three sampling concentrations, and the results are shown in Table 2. From the data in the table, we can see that the dust-removal and total-dust reduction efficiencies reach more than 90%. In the total dust dispersion test after dustfall, dust < 2.5 μm, which is the most harmful to humans, accounts for 63–85%. Although it accounts for a large proportion, the quantity is much reduced compared with that before treatment. 2.5–10 μm dust accounts for 10–35%, 10–20 μm dust accounts for 1–4%, and > 20 μm dust accounts for 0–1%. The dust control has a certain effect and can meet the expected requirements.

**Table 2:** Dust concentration and dust reduction efficiency.

Detection location	Sample number	Dust mass concentration before dust fall (mg/m^3^)		Dust mass concentration after dust fall (mg/m^3^)		Dust reduction efficiency (%)	
		Total coal dust	Respirable dust	Total coal dust	Respirable dust	Total coal dust	Respirable dust
3001 tail	1	125.6	33.9	3.7	1.7	97.05	94.98
3001 guide trough	2	125.1	36.5	3.6	1.6	97.12	95.61
3001 feed inlet	3	130.5	38.6	3.8	1.9	97.09	95.08
3002 middle section	4	119.5	27.4	3.6	1.6	96.98	94.16
3002 tail	5	115.4	30.6	3.0	1.8	97.40	94.12
3002 guide trough	6	125.9	40.5	3.9	1.9	96.90	95.31
Tape corridor junction	7	132.8	35.6	3.7	2.1	97.21	94.10
3002 feed inlet	8	120.7	32.6	3.4	1.7	97.18	94.79

**Figure 12:**
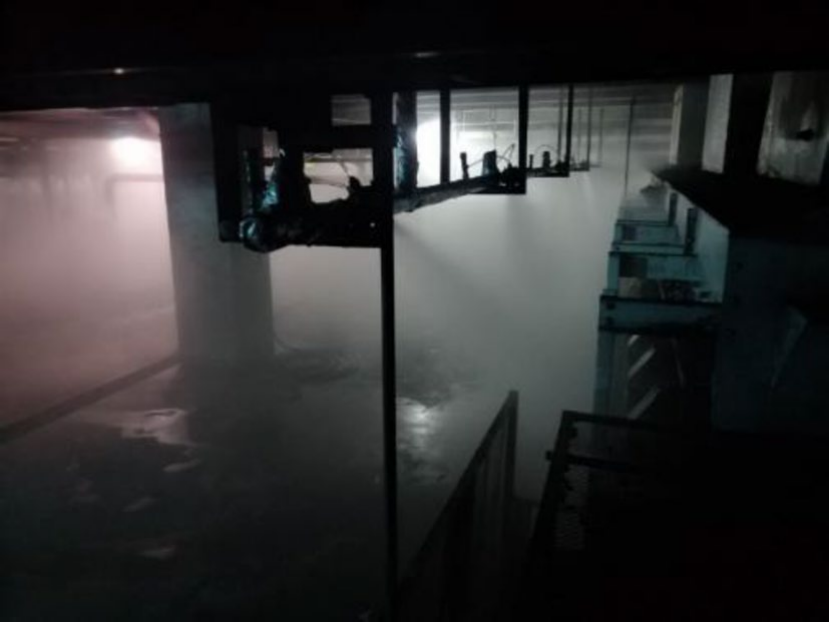
Dust reduction effect in the preparation workshop.

Figure 12 shows the dust reduction effect in the preparation workshop. It can be seen from the figure that the spray volume is large and fog is filled in the whole preparation workshop. Although the visibility around the fog curtain is poor at this time, the fog curtain can be dispersed after stopping the spray for 1–22 min, which does not affect emergency maintenance and repair. As far as the dust suppression function is concerned, the system can effectively destroy the distribution of the dust-containing airflow field, eliminate the dust migration and accumulation phenomenon when the double tape is working, collect the dust in the initial path of the dust source diffusion, and effectively cover and wrap the dust source point. Thus, diffusion is eliminated at the source, reaching an advanced level at home and abroad. The design requirements are realized, and the comprehensive dust reduction effect exceeds 90%.

## Conclusions

In this paper, a numerical model of the airflow-dust distribution of belts 3001 and 3002 on the first floor of the preparation workshop is established through simulation software. After detailed research, the obtained rules are verified for engineering applicability at the Huangyuchuan coal preparation plant, and spray dust reduction is arranged according to the site conditions. The test results of the system show that the dust reduction effect is good, and the specific analysis results are as follows:

1. Through the comprehensive comparison of the simulation results of the field and the air flow line, it can be seen that the air flow line gathering area will be formed between the two tapes and at the tail of the belt conveyor. There will be backflow on the right side of the 3001 tape and the left side of the 3002 tape, and the air flow velocity is generally below 0.6 m/s. The streamline velocity in the area close to the tape can reach more than 1 m/s.
2. By analyzing and comparing the migration state of particles with different particle sizes, it can be concluded that most of the dust particles larger than 10 μm are distributed in the range of 0–5 m on both sides of the tape; dust particles smaller than 10 μm have the widest diffusion range and spread to the entire workshop area.
3. The on-site spray dust suppression system achieves full coverage treatment of several areas with serious dust pollution at the tape inlet, machine tail, guide trough, and tape corridor connection with a good atomization effect. Compared with the changes in dust concentration before and after treatment, it is concluded that the reduction efficiency of dust and total dust is above 90%.
